# Views of Facial Attractiveness of Faces of Individuals With and Without an Intellectual Disability

**DOI:** 10.1111/jar.70161

**Published:** 2026-01-28

**Authors:** Harriet Whitby‐Tillott, Madeline Donnachie, Benedict Jones, Andrew Jahoda

**Affiliations:** ^1^ NHS Greater Glasgow Clyde Glasgow UK; ^2^ NHS Lanarkshire Lanarkshire UK; ^3^ University of Strathclyde Strathclyde UK; ^4^ University of Glasgow Glasgow UK

## Abstract

**Background:**

Little is known about people with intellectual disabilities' views of facial attractiveness and and how desirable they feel they are (self‐desirability).

**Method:**

Twenty‐four adults with intellectual disabilities and twenty‐five adults without disabilities were recruited from further education colleges and voluntary community organisations. Participants were asked to rate the attractiveness of facial images of typically developing individuals and individuals with Down syndrome. This was followed by a semi‐structured interview, exploring participants with intellectual disabilities' perceptions of self‐desirability.

**Results:**

The participants with intellectual disabilities gave consistent attractiveness ratings when evaluating images of people with an intellectual disability. They were also more likely, than not, to perceive themselves as desirable to those they found attractive.

**Conclusion:**

The findings suggest that people with intellectual disabilities make sophisticated intuitive judgments about the facial attractiveness of other people with and without intellectual disabilities, and remain positive about their own attractiveness.

## Lay Summary

- Finding a romantic partner is important part of most people's lives. However, few studies have asked people with intellectual disabilities about who they find attractive.
- We asked twenty five people with an intellectual disability to tell us how attractive they thought faces of individuals with and without intellectual disabilities were.
- Most study participants gave the same ratings of attractiveness to faces. They also thought that those they found attractive would be willing to ask them out on a date.
- The study participants share the same views about facial attractiveness, even if they are unable to say why they find the faces attractive. They also feel positive about themselves and their own attractiveness.

## 1 Background

Individuals with intellectual disabilities have the same sexual needs and desires for intimate relationships as those without intellectual disabilities (Parchomiuk 2022). Yet, little is known about people with intellectual disabilities' views of attractiveness and partner selection. Research by Bates et al. (2017) found that individuals with intellectual disabilities valued kindness, warmth, and companionship more than physical attractiveness, social status, intelligence, or financial security. While research by Mattila et al. (2017) found that participants with intellectual disabilities considered physical attractiveness to be an important feature of partner selection, they also emphasized the importance of shared interests, intimacy and empathy skills. However, selecting a partner is not solely based on someone's evaluation of others. It is also based on the judgments they receive from others about their own attractiveness (Clapton et al. 2018). Consequently, when someone selects a partner, they are also making a social comparison about their own ranking as potential partners.

Social Comparison Theory (Festinger 1954) proposes that self‐evaluation is achieved through comparison with others. People with intellectual disabilities can experience stigma, social isolation, or discrimination (Scior et al. 2022; Martinez‐Cao et al. 2021; Pelleboer‐Gunnink et al. 2021), which can negatively influence how they view themselves in relation to others (Paterson et al. 2012). However, not all individuals with intellectual disabilities experience stigma (Chien et al. 2020), and may be able to embrace their intellectual disability as a valued aspect of who they are (Kittelsaa 2014). Moreover, individuals with intellectual disabilities often make meaningful contributions to their communities and society, engaging in work, social, and volunteer activities that are highly valued by those around them (Arnold 2024). These contributions may themselves serve as important reference points within social comparison, fostering a sense of competence, belonging, and self‐worth. Chien et al. (2020) highlighted that supportive caregivers, inclusive communities, and affirming social relationships can foster adaptive self‐concepts in people with intellectual disabilities. However, it remains unknown how individuals with intellectual disabilities perceive their own facial appearance, which represents a critical gap for future research. This is particularly important, as interpersonal attractiveness plays a central role in psychosocial functioning, influencing not only opportunities for intimate relationships but also broader experiences of social inclusion (Prestia et al. 2002) and self‐esteem (Bale and Archer 2013). Understanding how individuals with intellectual disabilities perceive themselves and others in terms of attractiveness could therefore have important implications for their social engagement, self‐concept, and overall well‐being.

One approach to the study of attraction is by investigating facial preferences. Physical appearance, particularly the face, is a strong cue for discerning a person's attractiveness and determining partner choice (Gerhardstein and Anderson 2010). Specific facial features, such as symmetry and averageness, are often perceived as attractive (Jones et al. 2001; Little and Griffey 2020), with findings appearing consistent cross‐culturally (Apicella et al. 2007; Rhodes et al. 2001). Only one study by Donnachie et al. (2021) has applied a method commonly used in research on the general population to examine how individuals with intellectual disabilities perceive facial attractiveness. Participants with intellectual disabilities were asked to rate the attractiveness of standardised facial images. Despite the images being highly similar, with only subtle differences in features associated with attractiveness (e.g., symmetry), their ratings were remarkably consistent. This is a surprising finding, as previous research has shown that individuals with intellectual disabilities often struggle with facial recognition (Gawrylowicz et al. 2013) and interpreting emotional expressions (Matheson and Jahoda 2005). This suggests that individuals with intellectual disabilities can discern subtle differences in facial features linked to attractiveness. Furthermore, when comparing attractiveness ratings between individuals with and without intellectual disabilities, the study found a high level of agreement, implying a shared influence of cultural and societal beauty norms. Notably, individuals with intellectual disabilities were more likely than those without disabilities to perceive themselves as desirable to the facial images they found attractive. This finding indicates that, despite their often devalued social status, individuals with intellectual disabilities may maintain a positive sense of self‐worth.

Within facial imagery research there has been a movement towards using unmanipulated, naturalistic images that capture the normal variations in human faces, reflecting real‐life contexts (Jenkins et al. 2011). Donnachie et al.'s (2021) use of highly standardised images means that the findings do not offer insight into how people respond to more typical faces (Dawel et al. 2022). Moreover, Donnachie et al. (2021) did not include images of people with recognisable intellectual disabilities. Therefore, it remains unknown whether people with intellectual disabilities use similar visual cues when judging the attractiveness of facial images of people with a recognisable intellectual disability. This is of interest, as individuals with intellectual disabilities are more likely to interact with and have a romantic partnership with a person with an intellectual disability (Bates et al. 2017; Merrells et al. 2018).

### 1.1 Aims

This exploratory study builds on Donnachie et al.'s (2021) research on facial attractiveness by using a more naturalistic set of facial images, including images of people with a recognisable intellectual disability. It aimed to examine whether individuals with intellectual disabilities make consistent judgments about the attractiveness of naturalistic images of both typically developing individuals and people with a recognisable intellectual disability. A further aim was to investigate whether attractiveness ratings differ between individuals with and without intellectual disabilities when evaluating facial images of people both with and without an intellectual disability. In addition, the study aimed to explore how individuals with intellectual disabilities perceive their own attractiveness and view themselves as potential romantic partners, and how these self‐perceptions relate to their expectations of romantic interest from others, both with and without intellectual disabilities.

## 2 Method

### 2.1 Participants

Twenty‐four adults with intellectual disabilities and twenty‐five without intellectual disabilities were recruited. Participants with intellectual disabilities were recruited from community organisations and further education colleges offering specialist courses for people with intellectual disabilities. Those without intellectual disabilities included college students studying a range of health and social care courses, volunteers, and staff working in community organisations for people with intellectual disabilities. This comparison group was recruited using an opportunity sampling strategy, which was practical given the researcher's presence in these settings and participants' interest in the lives of people with intellectual disabilities.

All participants needed the ability to provide consent, have the appropriate receptive and expressive verbal ability to complete all the elements of the study and be aged 16–40 years. The age range was selected to align with prior research on attraction in both the general and intellectual disability populations (Bale and Archer 2013; Katsena and Dimdins 2015; Rojas et al. 2016). These life stages are particularly relevant to the emergence of romantic interest, attraction, and the desire for intimate relationships, as well as the potential for cohabitation and forming long‐term partnerships (Kaestle and Halpern 2007).

Suitable participants were identified with the help of college and support staff, who assessed if potential participants could (1) converse with others about sports, family, group activities, and so forth; (2) use complex sentences containing words like 'because' and 'but'; and (3) answer simple questions such as 'What is your name?' or 'What are you doing?' (Adaptive Behaviour Scale (ABS‐RC:2); Nihira et al. 1993). Participants were excluded if they had any sensory or mental health difficulties that would prevent them from completing the research tasks.

A cut‐off score of 75 on the Wechsler Abbreviated Scale of Intelligence—Second Edition (WASI‐II; Wechsler 2011), was applied for having a mild learning disability. The WASI‐II is a brief measure of IQ and does not provide the same accuracy as a full battery of ability tests. The test was administered by the first author, a psychologist who had received the appropriate training and authorisation to administer the assessment. Following data collection, two participants in the intellectual disabilities group were excluded from the analyses as their scores were above 75. Three participants recruited to the non‐intellectual disabilities group were also excluded from the analyses as their scores were below 75.

Table 1 shows the socio‐demographic characteristics of the 43 participants who took part in the study. Participants were divided into two groups: Group One, consisting of 21 individuals with intellectual disabilities, and Group Two, consisting of 22 individuals without intellectual disabilities. The intellectual disability group had more male (*n* = 14) than female (*n* = 7) participants. The opposite pattern was true for the non‐intellectual disability group, which had more female (*n* = 18) than male (*n* = 4) participants. The groups were of similar ages, with the majority aged from late teens to mid‐twenties. In terms of sexual orientation, 5 participants in the intellectual disability group identified as non‐heterosexual, compared to 9 participants in the non‐intellectual disability group. One participant with an intellectual disability said they did not know their sexual orientation. The majority of individuals with an intellectual disability identified as single (*n* = 14), whereas the majority without an intellectual disability stated they were in a relationship (*n* = 12). Most participants in both groups were living with their parents. Three participants with an intellectual disability did not provide a postcode and their socio‐economic status could be calculated. The two groups were similarly distributed across the deprivation categories, with the majority of participants in the most deprived category.

**TABLE 1:** Participant characteristics.

Variable	Intellectual disability group (*n* = 21) *n* (%)	Non‐intellectual disability group (*n* = 22) *n* (%)
Gender		
Female	7 (33.3%)	18 (81.8%)
Male	14 (66.7%)	4 (18.2%)
Age (years)		
Mean age (SD)	24.14 (8.06)	22.18 (5.24)
Range	23 (17–40 years)	14 (16–30 years)
Sexual orientation		
Heterosexual	15 (71.4%)	13 (59.1%)
Homosexual	2 (9.5%)	4 (18.2%)
Bisexual	3 (14.3%)	5 (22.7%)
Don't Know	1 (4.8%)	0
Relationship status		
Single	14 (66.7%)	10 (45.5%)
In a relationship	7 (33.3%)	12 (54.5%)
Living situation		
Living with parent/s	15 (71.4%)	12 (54.5%)
Living with grandparents	0	3 (13.6%)
Living with partner	0	3 (13.6%)
Living alone	4 (19%)	1 (4.6%)
Foster Care	2 (9.5%)	1 (4.6%)
Shared accommodation	0	1 (4.6%)
Living with children	0	1 (4.6%)
WASI‐II		
Mean (SD)	63.3 (7.48)	91.5 (9.57)
Range	22 (53–75)	33 (76–109)
SIMD quintiles	*n* = 18 (%)	*n* = 22 (%)
Most deprived 1	11 (52.4%)	15 (68.2%)
2	4 (19%)	3 (13.6%)
3	1 (4.8%)	3 (13.6%)
4	1 (4.8%)	0
Least deprived 5	1 (4.8%)	1 (4.6%)

*Note:* SIMD = Scottish Index Multiple Deprivation; WASI‐II = Wechsler Abbreviated Scale of Intelligence– Second Edition.

### 2.2 Experimental Tasks, Interview and Measures

The following data were gathered from participants in the order presented below.

#### 2.2.1 Background Information

Socio‐demographic information was gathered about each participant's age, gender, relationship status, living situation, sexual orientation, and socio‐economic status. Socio‐economic status was assessed using the Scottish Index of Multiple Deprivation (SIMD; Scottish Government 2016). SIMD uses a scale from one to five based on postcode, with one indicating the most deprived areas and five indicating the least deprived.

#### 2.2.2 Creation of the Attractiveness Rating Task

Participants were presented with a set of facial images of people with Down syndrome and a matched set of facial images of typically developing people. These were either images of males or females based on the participants' sexual preference. Individuals who identified as bisexual or were unsure of their sexual orientation were able to choose their preference of male or female faces.

There is no publicly available database which includes validated facial images of people with a recognisable intellectual disability. Consequently, the first step involved creating a set of facial images of people with a recognisable intellectual disability. A decision was made to use images of people with Down syndrome as it is a widely recognised genetic condition associated with intellectual disability and marked by distinct facial characteristics, including the epicanthic fold (Carr 1995). An expert in facial recognition research advised that 15 female and 15 male facial images were required to draw statistical conclusions. This number reflects established practice in facial attractiveness research, where around 12–20 stimuli per condition are typically sufficient to provide statistical variability while avoiding participant fatigue (Dawel et al. 2022; DeBruine 2004). Given the additional consideration of accessibility for participants with intellectual disabilities, this number was considered an appropriate balance between methodological rigor and practical feasibility.

An extensive search of freely available photo websites was conducted to find naturalistic images of people with Down syndrome, with varying levels of attractiveness. Images were selected where the individual appeared to be aged between 18 and 35, to reflect the age range of participants. Headshots were chosen where the individual posed front‐on to the camera with a neutral or slightly positive expression. To control for the possible effects of emotional cues on responses to faces, facial images displaying extreme emotions were excluded.

Once all searches were exhausted, a matched set of images was created of typically developing people with similar levels of attractiveness. This was achieved by searching stock photo websites for images of people with similar facial characteristics to each of the images in the first set. Matching was based on general facial characteristics and involved avoiding highly salient differences, including pose, head tilt, facial expression, eye color, hair color and style, facial hair, skin tone, and the presence of glasses or other facial paraphernalia.

A set of 26 female facial images and a set of 26 male images were produced. The images were then cropped to reveal only the individuals' heads and shoulders, aligned on pupil position, and set to a resolution of 1350 × 1800 pixels at 24‐bit (“true colour”) depth. People's hair and clothing remained visible in the images. The images were not standardised in terms of lighting, background, camera type, and angle. The images were printed to a size of 6 × 4 in, a standard photograph size, and laminated.

The images were mostly formal portrait photos of people with Down syndrome who were highly made up. To ensure it was possible to accurately identify the photographs, six independent raters were asked to decide whether or not the images depicted someone with Down syndrome. The images incorrectly identified as not having Down syndrome were excluded, along with the matched images without Down syndrome. Hence, the final sets of photographs consisted of 22 female and 22 male facial images of people with Down syndrome and 22 female and 22 male facial images of people without Down syndrome. Although the majority of images depicted white individuals of European descent, images of individuals from a range of other ethnic backgrounds were also represented.

#### 2.2.3 Attractiveness Rating Task

Following the approach taken by Donnachie et al. (2021), participants were asked to rate how attractive they viewed the facial images on a five‐point Likert scale; 1 = not at all, 2 = a wee bit, 3 = ok, 4 = quite, or 5 = very. The scale was presented on boxes, and the participants were asked to place the image in the box that corresponded with their answer. Blocks of increasing size were used alongside the boxes to visually represent the scale. To mitigate any potential order effects, the sequence of photo presentation, and the order in which facial images of people with and without Down syndrome were shown were alternated. Images of individuals with and without Down syndrome were presented in separate sets to avoid giving the impression that the aim was to compare the attractiveness of the two groups directly, allowing participants to provide independent judgements for each set.

#### 2.2.4 Semi‐Structured ‘Romantic Partner’ Interview

In keeping with Donnachie et al.'s (2021) study, a semi‐structured interview was used to gain insight into participants' views about attractiveness and how desirable they believe they are to others. Immediately, after rating all 22 images in a set, participants were asked to identify the image they considered most attractive. In the event of a tie, participants were asked to choose the image they would like to discuss further. The interview then focused on this selected image, with participants asked: “What made you think this person is attractive?”; “Do you think this person would ask you out on a date?” and “What do you think they would say if you asked them out on a date?”. Their reasons for these answers were then explored by asking, “Can you tell me the reasons that made you think that?” These questions were asked for both sets of facial images, with and without Down syndrome.

#### 2.2.5 Wechsler Abbreviated Scale of Intelligence—Second Edition (WASI‐II)

To determine if participants were in the appropriate groups, the WASI‐II was used to measure cognitive ability. The WASI‐II (Wechsler 2011) is an abbreviated version of the Wechsler Adult Intelligence Scale‐IV (Wechsler 2008) and serves as a measure of cognitive ability. The two‐subtest format was administered to determine if participants were in the appropriate groups. Psychometric properties of the WASI‐II include good to excellent test–retest reliability across subtests (0.83–0.94) and composite scores (0.90–0.96), a high level of internal reliability (0.90–0.92), and acceptable (0.71) to excellent (0.92) concurrent validity.

### 2.3 Procedure

The researcher met with groups of individuals who had expressed an interest in participating in the study at either their college or community organisation. During these meetings, the researcher discussed the easy‐read participant information sheet. For those who remained interested, at least 24 h later, the researcher arranged an individual session to go through the easy‐read consent process, ensuring that participants understood their role in the research, that participation was voluntary, and that they had the right to withdraw at any time. Once consent was obtained, the researcher completed the study with the participant.

The study took placein a private and confidential space, within their college or community service. Participants provided background information followed by the first attractiveness rating task of facial images. They then completed the semi‐structured interview about the highest‐rated image. The same order was followed for the second set of facial images. The WASI‐II was completed last to ensure that participants did not think they were being tested on their ability to get answers 'right' on the other tasks. At the end of the session, participants could provide feedback on their experiences of the tasks and ask any questions.

#### 2.3.1 Pilot Phase

Piloting was completed with two individuals with intellectual disabilities and two individuals without intellectual disabilities to examine the feasibility of the tasks. It was confirmed that both the attractiveness rating task and interviews were comprehensible to the participants and could be completed in an hour.

## 3 Ethics Statement

Research ethics approval was obtained from the University of Glasgow's College of Medical, Veterinary and Life Sciences Ethics Committee.

## 4 Analysis

### 4.1 Attractiveness Ratings

#### 4.1.1 Within Group Comparison

Cronbach's alpha was used to assess the consistency of attractiveness ratings made by participants with intellectual disabilities. This was calculated separately for images of people with Down syndrome and images of typically developing individuals. The same analysis was also conducted for the participants without intellectual disabilities. A Cronbach's alpha value of 0.70 or higher was considered to indicate acceptable internal consistency, reflecting consistent judgments across the different facial images within each group.

One heterosexual male with an intellectual disability rated all facial images as ‘not at all’ attractive due to being in a relationship. Consequently, his data were considered unreliable and excluded from the analysis.

#### 4.1.2 Between Group Comparison

Spearman's rho correlation was used to examine whether the attractiveness ratings made by participants with and without intellectual disabilities were similar for images of people with Down syndrome. The analysis was then repeated for facial images of typically developing individuals, allowing for a comparison of the two groups' judgments across both sets of facial images.

### 4.2 Perceptions of Self‐Desirability

#### 4.2.1 Quantitative Analyses

To explore how individuals with intellectual disabilities perceive their own attractiveness and romantic desirability, chi‐square tests of independence were conducted to examine differences in responses to the ‘dating scenario’ questions. These questions related to the images of the person with and without Down syndrome whom participants had rated as most attractive. Participants were asked: (1) Do you think this person would ask you out on a date? and (2) What do you think they would say if you asked them out on a date?

#### 4.2.2 Qualitative Analysis

To explore participants' reasons behind their expectations of romantic interest from others, recordings of the semi‐structured interviews were transcribed verbatim and analyzed using content analysis (Strauss 1987). Categories were created by extracting the different reasons participants provided in relation to acceptance or rejection in the dating scenario questions. If participants provided multiple reasons, their responses could fall into more than one category. After developing all the categories, an independent second rater, who was not involved in the study, was tasked with assigning the responses to the predefined categories. Agreement between the researcher and the second rater was assessed using Cohen's kappa coefficient, with strong agreement indicated by kappa values exceeding 0.80 for all questions. Disagreements were resolved through discussion. All analyses were two‐tailed, reflecting the exploratory nature of the study.

## 5 Results

### 5.1 Ratings of Attractiveness

The following findings show the attractiveness ratings by participants with and without an intellectual disability for the two sets of images. As noted previously, the gender distribution between the two groups was uneven and only a small number of non‐heterosexual participants were recruited. Consequently, the analyses were conducted separately for all participants who chose to rate female faces and those who chose to rate male faces. Research by Kranz and Ishai (2006) suggests that individuals rate the attractiveness of male and female faces similarly regardless of gender or sexual orientation, supporting the decision to combine these groups.

#### 5.1.1 Within Group Comparison

Agreement between attractiveness ratings was highly consistent for both participant groups. For individuals with intellectual disabilities, the Cronbach's alpha exceeded 0.80 for ratings of both male and female faces across images of individuals with and without Down syndrome. This suggests that individuals with intellectual disabilities made similar judgments about the characteristics that contribute to facial attractiveness. Similarly, for participants without intellectual disabilities, Cronbach's alpha also exceeded 0.80 across all face‐rating conditions, indicating strong internal consistency in attractiveness judgments within each group.

#### 5.1.2 Between Group Comparison

Mean ratings for each image were created by averaging the scores provided by participants within each respective group.

The study found a strong, statistically significant correlation between the two groups' attractiveness ratings for images of people with Down syndrome, with higher agreement for female faces (rho = 0.76, *p* < 0.001) than for male faces (rho = 0.57, *p* = 0.005). This suggests a shared perception of attractiveness when evaluating individuals with a recognisable intellectual disability. For images of people without Down syndrome, a significant correlation was observed in female faces (rho = 0.58, *p* = 0.005), but the association for male faces was weaker (rho = 0.35) and did not reach statistical significance (*p* = 0.111).

Caution needs to be exercised when interpreting these results, as the groups were unbalanced in terms of gender and sexuality.

### 5.2 Perceptions of Self‐Desirability

#### 5.2.1 Quantitative and Qualitative Analyses

Tables 2 and 3 show participants with intellectual disabilities' responses to the ‘dating scenario’ questions about the images of the person with and without Down syndrome whom participants rated as being most attractive. The analysis below includes responses from all participants with intellectual disabilities, regardless of whether they rated male or female faces.

**TABLE 2:** Reasons for being asked and not being asked out on a date.

Intellectual disability group overall response	Intellectual disability group most common reason for response	
*n* = 21 (%)	*n* (%)	
Images of people with Down syndrome		
Yes 15 (71.4%)	Physical attraction 3 (20%)	‘She would think I'm attractive’
	Personality 3 (20%)	‘Cause I'm kind and caring and loving’
No 6 (28.6%)	Physical attraction 2 (33.3%)	‘I don't think I'm quite that good looking’
	Confidence 1 (16.7%)	‘I don't have the social confidence’
Images of people without Down syndrome		
Yes 12 (57.2%)	Personality 4 (33.3%)	‘Cause I'm like very friendly and caring’
	Physical attraction 3 (25%)	‘I'm kind of attractive’
No 9 (42.8%)	Physical attraction 5 (55.6%)	“I just don't think I'm attractive”
	Own disability 1 (11.1%)	‘Cause of my disability’

**TABLE 3:** Responses for participant's offer of a date being accepted or rejected.

Intellectual disability group overall response *n* = 21 (%)	Intellectual disability group most common reason for response *n* (%)	
Images of people with Down syndrome		
Accepted 16 (76.2%)	Physical attraction 4 (25%)	‘I'm quite a handsome dude’
	Shared disability 3 (18.8%)	‘It looks like she has disabilities as well the same as me’
Rejected 5 (23.8%)	Confidence 2 (40%)	‘I get nervous and tongue twist my words’
	Physical attraction 1 (20%)	‘I think she would go out with a boy with better looks then me’
Images of people without Down syndrome		
Accepted 13 (61.9%)	Physical attraction 6 (46.2%)	‘I'm quite a handsome dude’
	Personality 5 (38.5%)	‘Cause I'm a nice person’
Rejected 8 (38.1%)	Physical attraction 3 (37.5%)	‘Because I'm not handsome’
	Type 2 (25%)	‘Maybe not be her type’

#### 5.2.2 Being Asked out on a Date

##### 5.2.2.1 Images of People With Down Syndrome

The results revealed a statistically significant difference (χ^2^ (1) = 3.86, *p* = 0.049), suggesting that individuals with intellectual disabilities are more likely to perceive themselves as being asked out by an attractive person with Down syndrome. Specifically, fifteen participants believed they would be asked out on a date, compared to six who did not.

##### 5.2.2.2 Images of People Without Down Syndrome

The results revealed that there was no statistically significant difference in participants with intellectual disabilities' beliefs around whether they would be asked out on a date, or not, by an attractive other without Down syndrome (χ^2^ (1) = 0.43, *p* = 0.051). Twelve participants believed they would be asked out on a date, compared to nine who did not.

##### 5.2.2.3 Reasons for ‘Yes’ Responses

Table 2 shows the reasons participants with intellectual disabilities gave for believing they would be asked on a date by an attractive other with and without Down syndrome. For both image sets, common reasons included their own ‘personality’ being viewed positively and being seen as ‘physically attractive’. Participants also attributed the reason for being asked on a date to the other person's positive personality traits, such as being ‘kind’ and ‘non‐judgemental’. Another reason for being asked out on a date was due to ‘similarities’, such as being of a similar age and engaging in the same social activities. Regarding the images of people with Down syndrome, two participants said they would be asked out on a date because they had a ‘shared disability’, with one individual explaining that they would have much in common. Conversely, one participant associated the other person's disability with being ‘flirtatious’ and lacking an understanding of right from wrong.

##### 5.2.2.4 Reasons for ‘no’ Responses

Table 2 details reasons participants with and without an intellectual disability provided for not being asked on a date out by an attractive other with and without Down syndrome. Irrespective of the facial image, the predominant reason cited by participants was ‘physical attraction’. Participants often felt they were not attractive enough to be asked out. Another reason, applicable to both sets of images, was a lack of ‘confidence,’ with participants describing themselves as ‘shy’. One participant attributed their ‘own disability’ as a reason for not being asked out by an attractive other without Down syndrome.

#### 5.2.3 Offer of a Date

##### 5.2.3.1 Images of People With Down Syndrome

The results revealed a statistically significant difference (χ^2^ (1) = 5.76, *p* = 0.02), suggesting that individuals with intellectual disabilities are more likely to perceive that their offer of a date would be accepted by an attractive person with Down syndrome. Specifically, sixteen participants believed their date offer would be accepted, compared to five who believed it would be rejected.

##### 5.2.3.2 Images of People Without Down Syndrome

The results revealed that there was no statistically significant difference in participants with intellectual disabilities' beliefs around whether or not their offer of a date would be accepted by an attractive other without Down syndrome (χ^2^ (1) = 1.19, *p* = 0.28). Thirteen participants believed their date offer would be accepted, compared to eight who believed it would be rejected.

##### 5.2.3.3 Reasons for ‘Accepted’ Responses

Table 3 shows the most common reason participants gave for a date being accepted by the person with and without Down syndrome was ‘physical attraction’. Participants also thought they would be asked on a date because of their personality. Regarding the images of people with Down syndrome, three participants cited a ‘shared disability’ as a reason, noting that it makes it ‘fair’ and ‘easier to get along’. Interestingly, one participant noted that their offer of a date would be accepted as the person with Down syndrome would not understand ‘what is right’.

##### 5.2.3.4 Reasons for ‘Rejected’ Response

Table 3 shows the common reasons participants provided for their offer of a date being rejected by an attractive individual, with and without Down syndrome. Irrespective of the facial image, participants gave ‘physical attraction’ as a reason and expressed a self‐perceived lack of attractiveness. A lack of confidence in asking someone out was also mentioned for both image sets, as well as the notion of being the wrong ‘type’ or feeling that they did not fit the perceived preferences of the other person.

## 6 Discussion

Consistent with Donnachie et al. (2021), participants with intellectual disabilities showed a high level of agreement in their attractiveness ratings of typically developing individuals. Moreover, participants also showed a high level of agreement in their attractiveness ratings of people with a recognisable intellectual disability. Despite research suggesting that individuals with intellectual disabilities often struggle with facial recognition (Gawrylowicz et al. 2013) and interpreting emotional expressions (Matheson and Jahoda 2005), the participants exhibited consistent judgments of facial attractiveness across images of individuals with and without an intellectual disability. Research has shown that certain facial features are universally considered attractive across people of different ages and ethnicities (Mengelkoch et al. 2022). The current findings suggest that individuals with intellectual disabilities may rely on these same cues when judging the attractiveness of faces, including those with a recognisable intellectual disability. However, due to the unstandardised nature of the images, the specific features influencing these judgments could not be determined.

Additionally, a positive association was found between the attractiveness ratings of individuals with and without intellectual disabilities, when evaluating facial images of people with and without Down syndrome. This suggests that both groups use similar cues to assess facial attractiveness. However, caution is needed when interpreting this comparison because the groups differed in key characteristics such as gender and sexuality, which were not matched across participants with and without intellectual disabilities.

In line with Donnachie et al.'s (2021) findings, participants in this study were likely to view themselves as desirable to those they found attractive. The participants with an intellectual disability were likely to believe they would be invited on a date and have their offer accepted by an attractive person without an intellectual disability. Consequently, participants in this study demonstrated a positive sense of self‐worth. This is particularly interesting given prior research indicating that individuals with intellectual disabilities often face rejection and mockery from non‐disabled individuals when using online dating platforms (Martino and Kinitz 2022; McCarthy et al. 2020).

A proportion of participants with intellectual disabilities were likely to perceive themselves as desirable to an attractive individual with Down syndrome. This may reflect the greater opportunities they have to form social connections and romantic relationships with others with an intellectual disability (Bates et al. 2017; Merrells et al. 2018). Three participants specifically cited their shared disability as a reason for being viewed as desirable by someone with Down syndrome.

Interestingly, only one participant attributed their disability as a reason for not being asked out by an attractive other without Down syndrome. Instead, their reasons for rejection seem to focus on more universal factors, such as physical attractiveness, confidence and compatibility with the other person's ‘type’. However, it is worth considering whether the notion of ‘type’ might implicitly include disability‐related perceptions. Consequently, further exploration is required to better understand how individuals' self‐perception of disability influences how desirable they see themselves as partners.

## 7 Strengths and Limitations

A notable strength of this study is that it is one of the first to incorporate naturalistic images of faces, including those with visible intellectual disabilities, into attractiveness research. Previous studies in this area have predominantly relied on computer‐generated and highly standardised images, which lack ecological validity. In contrast, the use of naturalistic images in the present study provides a more authentic representation of the diversity of faces encountered in real‐world social interactions.

However, the use of non‐standardised images prevented precise control over specific facial cues. As a result, it was not possible to determine which particular facial features influenced participants' attractiveness judgments, limiting the ability to draw causal inferences.

A major limitation was the small sample size, which included an uneven distribution of genders between the two groups. Consequently, meaningful comparisons between groups could not be made. In addition, there was notable heterogeneity within both samples in terms of sexual orientation, relationship status, and socio‐economic background. These factors may have influenced participants' views on attractiveness and self‐perception, and caution needs to be exercised when interpreting the study findings.

Furthermore, the relatively small sample of participants with intellectual disabilities meant that some responses in the semi‐structured interviews were represented by only a single individual. However, qualitative findings of this nature are not expected to be generalisable and the insights gained from this study should be viewed as a contribution towards broadening the scope of research on facial attractiveness and self‐desirability in individuals with intellectual disabilities.

The present study focused on the responses of participants with intellectual disabilities. Participants without intellectual disabilities expressed discomfort when asked to make judgments about the prospect of a romantic relationship with an individual with an intellectual disability. Given the legal and ethical considerations surrounding the capacity of individuals with intellectual disabilities, it is understandable that participants may have felt uneasy discussing this topic. As noted by Donnachie et al. (2021), participants without intellectual disabilities may also have been influenced by social desirability, adjusting their responses to appear more modest or socially appropriate in front of the researcher. Additionally, the use of naturalistic facial images, including those of people with a visible intellectual disability, may have shaped how participants engaged with the task, differing from previous studies that typically used computer‐generated faces. Participants may have felt a stronger personal connection to the images and, as a result, been more aware of how their judgments reflected on their attitudes towards others, particularly those with intellectual disabilities. Furthermore, many participants were volunteers or staff working in community organizations supporting people with intellectual disabilities. This prior familiarity may have influenced their judgments of the attractiveness of faces with Down syndrome, representing a potential limitation, as prior studies suggest that greater exposure to individuals with Down syndrome is associated with more positive attitudes (Enea‐Drapeau et al. 2012). Taken together, these factors highlight an important methodological consideration in attractiveness research, as participants' responses are likely shaped by their personal biases, familiarity, and awareness of social norms, which limits the extent to which such judgments can be considered to be objective.

## 8 Further Research

Future research should aim to include a more representative sample, encompassing individuals from diverse backgrounds and cultural perspectives.

Only one participant in the intellectual disability group had Down syndrome, meaning that it was not possible to evaluate whether individuals with Down syndrome judge Down syndrome faces differently from those without Down syndrome. This would be of particular interest for future research, as studies have shown that facial resemblance can influence judgements of attractiveness (DeBruine 2004).

An additional consideration is the typicality of the Down syndrome facial features in the images. Previous research indicates that the degree to which a face displays features characteristic of Down syndrome can influence attitudes and stereotyping (Enea‐Drapeau et al. 2012; Enea‐Drapeau et al. 2014). In the current study, the naturalistic images of individuals with Down syndrome were not systematically rated for feature typicality. As a result, it is not possible to determine whether differences in perceived attractiveness were influenced by the distinctiveness of Down syndrome features, highlighting an important area for future research.

This exploratory study was based on hypothetical situations. Future research should investigate participants with intellectual disabilities' actual experiences of being asked on a date and initiating romantic advances, as well as how they cope with rejection and its impact on their self‐concept. Furthermore, the study did not directly explore participants' experiences and views on dating individuals with intellectual disabilities. Investigating these perspectives and experiences could provide deeper insights into how individuals with intellectual disabilities perceive romantic relationships, including how their self‐perception of disability influences these views.

## 9 Conclusions

Good quality social and personal relationships are associated with better physical and mental health (McCarthy et al. 2020). This study has helped enhance our understanding of individuals with intellectual disabilities' views of attractiveness, and their perceptions of self‐desirability. However, it is unclear whether these findings apply to everyday situations. This highlights the importance of carers and support professionals engaging in open conversations with individuals with intellectual disabilities about romantic relationships. Traditional sex education often focuses solely on physical aspects, but it is equally important to address the emotional and social dimensions of relationships. By doing so, caregivers can help promote a more positive and holistic understanding of sexual expression and relationships.

## Funding

The authors have nothing to report.

## Ethics Statement

Ethical approval was obtained from the University of Glasgow College of Medical, Veterinary and Life Sciences Ethics Committee.

## Conflicts of Interest

The authors declare no conflicts of interest.

## Data Availability

The data that support the findings of this study are available on request from the corresponding author. The data are not publicly available due to privacy or ethical restrictions.
